# Transcriptome analysis of the anhydrobiotic cell line Pv11 infers the mechanism of desiccation tolerance and recovery

**DOI:** 10.1038/s41598-018-36124-6

**Published:** 2018-12-18

**Authors:** Takahiro G. Yamada, Yoshitaka Suetsugu, Ruslan Deviatiiarov, Oleg Gusev, Richard Cornette, Alexander Nesmelov, Noriko Hiroi, Takahiro Kikawada, Akira Funahashi

**Affiliations:** 1 Department of Biosciences and Informatics, Keio University, Yokohama, Kanagawa 223-8522 Japan; 2 Institute of Agrobiological Sciences, National Agriculture and Food Research Organization (NARO), Tsukuba, Ibaraki 305-8634 Japan; 3 Kazan Federal University, Kazan, Tatarstan 420008 Russia; 4 RIKEN, Yokohama, Kanagawa 230-0045 Japan; 5 Faculty of Pharmaceutical Science, Sanyo-Onoda City University, Sanyo-Onoda, Yamaguchi 756-0884 Japan; 6 Graduate School of Frontier Sciences, The University of Tokyo, Kashiwa, Chiba 277-8562 Japan

## Abstract

The larvae of the African midge, *Polypedilum vanderplanki*, can enter an ametabolic state called anhydrobiosis to overcome fatal desiccation stress. The Pv11 cell line, derived from *P. vanderplanki* embryo, shows desiccation tolerance when treated with trehalose before desiccation and resumes proliferation after rehydration. However, the molecular mechanisms of this desiccation tolerance remain unknown. Here, we performed high-throughput CAGE-seq of mRNA and a differentially expressed gene analysis in trehalose-treated, desiccated, and rehydrated Pv11 cells, followed by gene ontology analysis of the identified differentially expressed genes. We detected differentially expressed genes after trehalose treatment involved in various stress responses, detoxification of harmful chemicals, and regulation of oxidoreduction that were upregulated. In the desiccation phase, L-isoaspartyl methyltransferase and heat shock proteins were upregulated and ribosomal proteins were downregulated. Analysis of differentially expressed genes during rehydration supported the notion that homologous recombination, nucleotide excision repair, and non-homologous recombination were involved in the recovery process. This study provides initial insights into the molecular mechanisms underlying the extreme desiccation tolerance of Pv11 cells.

## Introduction

Desiccation stress, the loss of essential water, can be fatal. To tolerate desiccation stress, various organisms, such as rotifers, tardigrades, nematodes, plants, and larvae of the African midge *Polypedilum vanderplanki*, enter an ametabolic state called anhydrobiosis^1,2^ and survive even if more than 99% of body water is lost^3^. According to the water replacement hypothesis, a compatible solute, such as trehalose, protects phospholipid membranes and intracellular biological molecules and ensures their preservation under desiccation^3,4^. Prolonged desiccation can lead to serious oxidative stress. For example, in the moss *Fontinalis antipyretica* an increase in the production of reactive oxygen species (ROS) is associated with dehydration^5,6^. Protein oxidation in the dehydrated cells of the yeast *Saccharomyces cerevisiae* is 10 times that of hydrated cells^5,7^. Thioredoxins (TRXs) remove harmful ROS and protect cells from ROS-induced damage^5,7^. The genome of *P. vanderplanki* has a paralogous gene cluster for TRXs^8^. These TRXs are upregulated by dehydration, and *P. vanderplanki* becomes tolerant to ROS-induced damage^8^. Upon rehydration, the anhydrobiotes return to active life.

In 2002, the Pv11 cell line was established as an embryonic cell culture from *P. vanderplanki*^9^. Desiccation tolerance of Pv11 cells is induced by treatment with culture medium containing 600 mM trehalose for 48 h. Even after dehydration in a desiccator (<10% relative humidity) for 12 days and rehydration for 1 h, trehalose-treated Pv11 cells are able to resume proliferation^10^, whereas other insect cell lines (Sf9, BmN-4, AeAl-2, AnCu-35, and S2) do not. Pv11 cells are considered the only desiccation-tolerant insect cell line able to restore the regular cell cycle after rehydration, but it is unclear whether rehydrated Pv11 cells genuinely return to the same state. Analysis of gene expression patterns of Pv11 cells during trehalose treatment, desiccation, and rehydration may help to elucidate which genes are required to avoid cell death and return Pv11 cells from anhydrobiosis to the normal state.

In this study, we performed high-throughput CAGE-seq of mRNA and a differentially expressed gene (DEG) analysis in trehalose-treated, desiccated, and rehydrated Pv11 cells, followed by gene ontology (GO) analysis of the identified DEGs. To the best of our knowledge, this is the first report of a comprehensive DEG analysis using CAGE-seq data for Pv11 cells that infers a putative mechanism of their avoidance of cell death and recovery from anhydrobiosis.

## Results

### Gene expression analysis by CAGE-seq

**Figure 1:**
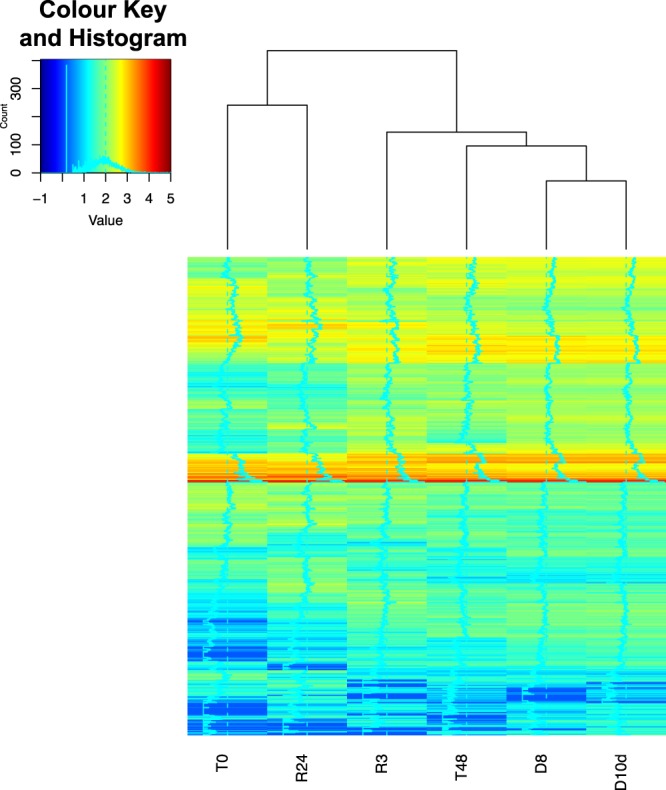
Hierarchical clustering based on tags per million (TPM) for each sample (distance: Euclidean). In the Colour Key and Histogram inset, the horizontal axis shows $lo{g}_{10}(TPM+minimum\,TPM\,excluding\,\mathrm{0)}$ and the vertical axis shows the number of DEGs. Genes were selected when they were detected as DEGs in at least one pair of samples.

To detect genes potentially related to desiccation tolerance and successful recovery after rehydration, we analysed DEGs in control Pv11 cells (T0) and at different stages of anhydrobiosis: trehalose treatment for 48 h (T48), desiccation for 8 h or 10 days (D8 and D10d), and rehydration of D10d cells for 3 or 24 h (R3, R24). We compared each pair of samples (false discovery rate [FDR] < 0.05; Table 1, Supplementary Data 1) and visualized the overall and differential expression of genes for each pair of samples as M-A plots (Supplementary Fig. S1). All of the median M values of non-DEGs were closer to 0 than those of DEGs; no DEGs were detected between D8 and D10d because metabolism of Pv11 cells completely stops under both these conditions. Samples were clustered using a hierarchical clustering algorithm (Fig. 1). The patterns of mRNA expression in the completely desiccated samples, D8 and D10d, were the most different from that of the normally cultured samples (T0). The expression patterns of R3 and R24 gradually approached that of T0.

**Table 1:** Numbers of differentially expressed genes.

	T0	T48	D8	D10d	R3
T48	384				
D8	813	37			
D10d	974	44	0		
R3	249	26	25	14	
R24	327	471	704	784	72

Each sample was analysed in biological triplicate, and the gene was considered a DEG at FDR < 0.05 (Benjamini-Hochberg method). We analysed DEGs in control Pv11 cells (T0) and at different stages of anhydrobiosis: trehalose treatment for 48 h (T48), desiccation for 8 h or 10 days (D8 and D10d), and rehydration of D10d cells for 3 or 24 h (R3, R24).

### Gene Ontology analysis of DEGs

We expected that DEGs between T0 and T48 would be genes functionally important for desiccation tolerance. We detected 299 upregulated and 85 downregulated DEGs between T0 and T48 (Supplementary Data 1). No enriched GOs for DEGs between T0 and T48 were detected (FDR ≥ 0.05, Supplementary Data 2). Therefore, we analysed the frequency of GOs among these DEGs. The first and second most frequent GOs were GO:0008152 (metabolic process) and GO:0055114 (oxidation-reduction process) in the biological process category, GO:0005524 (ATP-binding) and GO:0008270 (zinc ion binding) in the molecular function category, and GO:0005634 (nucleus) and GO:0016021 (integral component of membrane) in the cellular component category. Because metabolism and oxidoreduction are crucial for dehydration^3,8^, the genes with these GOs may be important for the induction of the desiccation tolerance mechanism.

Between T0 and D8, we detected 540 upregulated and 273 downregulated DEGs (Supplementary Data 1). GO analysis of these 813 DEGs identified three significantly enriched GOs: GO:0005484 (SNAP receptor activity), GO:0004719 (protein-L-isoaspartate (D-aspartate) O-methyltransferase activity), and GO:0006479 (protein methylation) (Supplementary Data 2). Almost all of the genes with GO:0004719 and GO:0006479 were upregulated in D8 and were similar to genes encoding protein L-isoaspartyl methyltransferase (PIMT) (BLASTX, E-value < 1.0E-05), which is an enzyme that recognizes and repairs damaged L-isoaspartyl groups in proteins^11^. PIMTs have been previously reported as important genes for desiccation tolerance in *P. vanderplanki* larvae.

Between T0 and D10d, we detected 624 upregulated and 350 downregulated DEGs (Supplementary Data 1). GO analysis of these 974 DEGs identified three significantly enriched GOs: GO:0006412 (translation), GO:0003735 (structural constituent of ribosome), and GO:0019843 (rRNA binding) (Supplementary Data 2). Most of the genes with these GOs were downregulated in D10d and were similar to genes encoding ribosomal proteins (BLASTX, E-value < 1.0E-05). Ribosomal proteins are essential for protein translation, which suggests protein translation in Pv11 cells in the dehydrated state is highly suppressed.

Between T48 and D8, we detected 27 upregulated and 10 downregulated DEGs (Supplementary Data 1). GO analysis of these 37 DEGs identified 25 significantly enriched GOs (Supplementary Data 2). The enriched GOs with the lowest FDRs were GO:0005212 (structural constituent of eye lens) and GO:0006376 (mRNA splice site selection). The DEGs with GO:0005212 were similar to genes encoding heat shock proteins (HSPs) (BLASTX, E-value < 1.0E-05). HSPs chaperone proteins that refold stress-damaged proteins, and HSPs were reported as an important proteins for desiccation tolerance in *P. vanderplanki* larvae^12^.

Between T48 and D10d, we detected 32 upregulated and 12 downregulated DEGs (Supplementary Data 1). GO analysis of these 44 DEGs identified 36 significantly enriched GOs and the GO with the lowest FDR was GO:0005212 (structural constituent of eye lens) (Supplementary Data 2). This GO was also identified in the comparison of T48 and D8.

This analysis revealed that genes associated with the recovery of damaged proteins (i.e., genes encoding PIMTs and HSPs) were significantly upregulated and genes essential for protein translation were significantly downregulated in the desiccation phase. Furthermore, no DEGs were identified between D8 and D10d. This suggests that, in the desiccation phase, protein translation is highly suppressed and that transcripts synthesized in the pretreatment stage are maintained.

**Figure 2:**
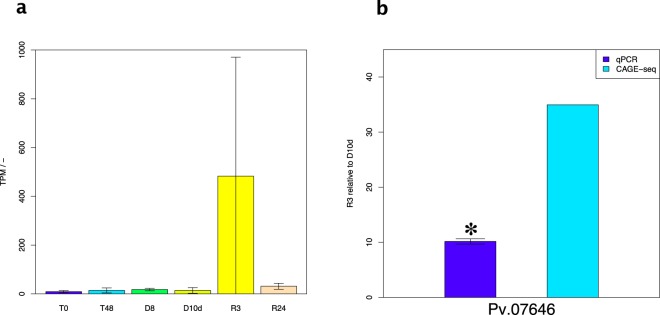
Expression of Pv.07646, as quantified by RNA-seq and RT qPCR. (**a**) Expression of Pv.07646, annotated by GO:0036055 (protein-succinyllysine desuccinylase activity) and GO:0036049 (peptidyl-lysine desuccinylation). Horizontal axis shows sample names. TPM, tags per million. Data are mean ± SD, n = 3. (**b**) Fold change from R3 to D10d, as determined by RT qPCR and CAGE-seq. *Significant change by Welch’s t-test (*p*-value < 0.05).

DEGs between D10d and R3 are likely to be functionally important for recovery from anhydrobiosis. In recovering cells, we detected 7 upregulated and 7 downregulated genes (Supplementary Tables S1, S2, Data 1). GO analysis of these 14 genes identified 8 significantly enriched GOs (Supplementary Data 2). The gene Pv.01867 was annotated by GO:0048763 (calcium-induced calcium release activity) and GO:0032237 (activation of store-operated calcium channel activity). Pv.04558 was annotated by GO:0030478 (actin cap) and GO:0070252 (actin-mediated cell contraction). Pv.07646 was annotated by GO:0036055 (protein-succinyllysine desuccinylase activity), GO:0036049 (peptidyllysine desuccinylation), GO:0036054 (protein-malonyllysine demalonylase activity), and GO:0036047 (peptidyllysine demalonylation). Pv.07646 was the most significant among the DEGs identified between D10d and R3; it was upregulated specifically in the R3 stage in some biological replicates (35 times vs. D10d and 15.5 times vs. R24; Fig. 2a). RT-qPCR confirmed significant up-regulation of Pv.07646 (Fig. 2b, Welch’s t-test: *p*-value < 0.05). The two other genes were not specifically upregulated in R3 (Pv.01867: 4.93 times for R3 vs. D10d and 0.99 for R3 vs. R24; Pv.04558: 0.12 for R3 vs. D10d and 0.11 for R3 vs. R24; Supplementary Fig. S2).

### Analysis of desiccation tolerance mechanism

#### Hierarchical clustering of DEGs between T0 and T48

**Figure 3:**
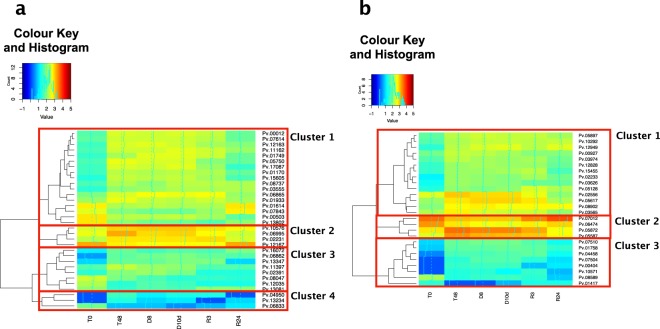
Hierarchical clustering of differentially expressed genes (DEGs) with (**a**) GO:0008152 (metabolic process) and (**b**) GO:0055114 (oxidation-reduction process). In the Colour Key and Histogram insets, the horizontal axes show $lo{g}_{10}(TPM+minimum\,TPM\,excluding\,\mathrm{0)}$ and the vertical axes show the number of DEGs.

To reveal the biological function of the genes that participate in the induction of desiccation tolerance in Pv11 cells, we focused on the DEGs between T0 and T48 with GO:0008152 (metabolic process; 32 DEGs) and GO:0055114 (oxidation-reduction process; 26 DEGs). To categorize these genes, we performed hierarchical clustering based on the data for each treatment. The expression patterns of GO:0008152 DEGs were classified into 4 clusters (Fig. 3a, Supplementary Table S3). During desiccation after trehalose treatment, some DEGs were expressed consistently (Cluster 1, Supplementary Fig. S3), but many were downregulated (Clusters 2–4, Supplementary Fig. S3). The expression patterns of DEGs with GO:0055114 were classified into 3 clusters (Fig. 3b, Supplementary Table S4). During desiccation, the expression of DEGs in clusters 1 and 3 was maintained or upregulated, whereas DEGs in cluster 2 were comparatively downregulated (Supplementary Fig. S4). The differences in the expression patterns of DEGs with the same GO during trehalose treatment, desiccation, and rehydration suggest different roles of these genes that enable Pv11 cells to acquire desiccation tolerance.

#### Various protection systems in the induction of desiccation tolerance

To clarify the contributions of DEGs to the induction of desiccation tolerance, we focused on the functions of genes with GO:0008152 and GO:0055114 identified by BLASTX searches. Pv.03555 (GO:0008152) has the highest similarity to the nonsense-mediated mRNA decay (NMD) protein (Supplementary Fig. S5), which distinguishes mRNA containing a premature stop codon and induces its degradation^13^. Pv.06995 (GO:0008152) has the highest similarity to glutathione S-transferase (GST) (Supplementary Fig. S6). GST is critical for protection against lipid peroxidation and for detoxification of oxidized lipids^14^. In *Arabidopsis thaliana*, upregulation of the gene encoding GST protects from drought stress^15^. Pv.03555 and Pv.06995 were significantly upregulated in T48 in comparison with T0, indicating that the expression of various stress response genes was induced by trehalose treatment in preparation for stress caused by dehydration. Pv.11397 (GO:0008152) has the highest similarity to UDP-glucuronosyltransferase (UGT) (Supplementary Fig. S7), which catalyses the glucuronidation of toxic chemicals such as bilirubin, phenols, amines, and carboxylic acids^16^. Therefore, Pv.11397 is likely to contribute to the detoxification of some harmful chemicals during induction of desiccation tolerance. In *Caenorhabditis elegans*, UGT is upregulated 6.5 fold after dehydration^17^. Pv.04950 (GO:0008152) has the highest similarity to carboxylesterase (Supplementary Fig. S8). Carboxylesterases catalyse the hydrolysis of xenobiotics with ester and amide groups^18^. Pv.11397 and Pv.04950 were significantly upregulated in T48 in comparison with T0. On the basis of the functions of these genes, we assume that their upregulation allows removal of harmful chemicals.

Many GO:0008152 genes were downregulated after dehydration, likely because Pv11 cells enter an ametabolic state after dehydration and metabolism-related genes may become unessential. On the other hand, the expression of GO:0008152 DEGs in Cluster 1 (Fig. 3) was maintained during dehydration. These DEGs included Pv.03555, which has the highest similarity to the NMD protein (as described above); Pv.08737, which has the highest similarity to ATP-dependent RNA helicase (Supplementary Fig. S9); and Pv.12163, which has the highest similarity to DEAD box ATP-dependent RNA helicase (Supplementary Fig. S10). Both NMD protein and RNA helicases act in the clearance of failed mRNA^19^. Therefore, the corresponding proteins of *P. vanderplanki* likely allow efficient elimination of imperfect mRNA that may result from strongly oxidizing conditions during dehydration.

Pv.00404, Pv.06474, Pv.07504, and Pv.07510 (oxidation-reduction process) have the highest similarity to TRXs and have been reported as PvTrx1-1, PvTrx3, PvTrx7, and PvTrx10, respectively^8^. These genes are upregulated after dehydration in the larvae of *P. vanderplanki*^8^. We found that these genes were significantly upregulated in T48 in comparison with T0. These findings indicate that oxidoreduction is part of the mechanism for the induction of desiccation tolerance and show an analogy between Pv11 cells and larvae of *P. vanderplanki*.

### Analysis of the mechanisms of recovery from anhydrobiosis

#### Domain structure analysis of the Pv.07646 gene

The alignment of Pv.07646 and its putative homolog SNF histone linker PHD ring helicase is shown in Supplementary Fig. S11. Histone Lys residues are succinylated and malonylated^20^. To recognize succinylated or malonylated Lys residues, the enzyme involved in desuccinylation or demalonylation needs to bind both the histone and DNA. Pv.07646 satisfies these requirements because it has a RING domain, which may be involved in protein-protein interactions, and a nucleotide binding site. However, Pv.07646 has no sequence similarity to the SIRT5 region, which is responsible for desuccinylation and demalonylation, suggesting that Pv.07646 may not directly catalyse these reactions. Pv.07646 has the highest similarity to the DNA-repair protein RAD16. The alignment analysis showed that Pv.07646 has sequences homologous to the HepA domain and the ATP-binding site of RAD16 (E-value, 0.0; Supplementary Fig. S12, Table S2). Rad16 forms a complex with Rad7 and binds DNA in an ATP-dependent manner^21^. When the HepA region of Rad16 is deactivated and DNA is damaged by UV radiation, the survival rate of *Escherichia coli* is significantly decreased^22^. These results suggest that Pv.07646 may act in DNA repair.

#### Differential expression of genes related to DNA repair in anhydrobiosis

Pv.07646 was significantly upregulated in R3, and this gene has sequence similarity to Rad16, which is important for DNA repair. This suggests that DNA repair could be critical for recovery from anhydrobiosis in Pv11 cells. Previous studies have revealed the genes related to several DNA repair systems, namely, homologous recombination (HR), nucleotide excision repair (NER), mismatch excision repair (MMR), and non-homologous end joining (NHEJ)^23,24^. We therefore comprehensively analysed the differential expression of these genes in the Pv11 cells (Table 2, Supplementary Data 3). For genes associated with HR, Pv.04176, which has sequence similarity to Rad51B, Rad51, XRCC3, and DMC1 which were homolog of Rad51 was upregulated in D10d, suggesting that DNA repair by HR was active in dehydration. For genes associated with NER, Pv.05115, Pv.05985, and Pv.01131, which have sequence similarity to ERCC3(XPB), GTF2H4, and GTF2H2, respectively, were significantly upregulated in D8 and D10d. In addition, Pv.10801 and Pv.01957, which have sequence similarity to XPA and CCNH, were significantly upregulated in R24. These results suggest that NER was active in dehydration and rehydration. For genes associated with NHEJ, Pv.10272, which has sequence similarity to XRCC5(Ku80), and Pv.15195, which has sequence similarity to LIG4, were significantly upregulated in D8 and D10d. Pv.15195 was also significantly upregulated in T48. These results suggest that NHEJ was active in pretreatment and dehydration. No significant upregulation of genes associated with MMR were detected. Thus, these results suggest that HR, NER, and NHEJ, but not MMR, could be important for desiccation tolerance and the process of recovery from anhydrobiosis in Pv11 cells.

**Table 2:** Differentially expressed genes with sequence similarity to the genes associated with homologous recombination, nucleotide excision repair, and non-homologous end joining (BLASTX E-value < 1.0E15).

Gene Name	Accession Number	Pv Gene ID	E.value	Pv11			*P. vanderplanki*		Pn Gene ID	E.value	*P. nubifer*
				Pretreatment	Dehydration	Rehydration	Dehydration	Rehydration			Dehydration
Homologous Recombination											
RAD51B	NM_002877.5	Pv.04176	1.40E-23	—	T0vsD10d	—	—	—	Pn.00701	4.89E-25	—
RAD50	NM_005732.3	Pv.02239	3.30E-127	—	—	—	—	D48vsR3	Pn.14177	1.60E-122	—
RAD51	NM_002875.4	Pv.04176	2.88E-150	—	T0vsD10d	—	—	—	Pn.00701	1.33E-153	—
XRCC3	NM_005432.3	Pv.04176	1.30E-16	—	T0vsD10d	—	—	—	Pn.13575	4.00E-22	—
DMC1	NM_007068.3	Pv.04176	1.27E-104	—	T0vsD10d	—	—	—	Pn.00701	9.47E-105	—
Nucleotide Excision Repair											
XPA	NM_000380.3	Pv.10801	1.00E-57	—	—	D10dvsR24	—	D48vsR3	Pn.08841	5.86E-49	—
ERCC3(XPB)	NM_000122.1	Pv.05115	0.0	—	T0vsD8, T0vsD10d	—	—	—	Pn.12708	0.0	—
GTF2H4	NM_001517.4	Pv.05985	5.29E-147	—	T0vsD10d	—	—	D48vsR24	Pn.12750	1.07E-148	—
GTF2H2	NM_001515.3	Pv.01131	7.35E-128	—	T0vsD8	—	—	—	Pn.09211	5.95E-130	—
CCNH	NM_001239.3	Pv.01957	2.63E-69	—	—	D10dvsR24	—	—	Pn.07710	3.09E-71	—
Non-Homologous End Joining											
XRCC6(Ku70)	NM_001469.4	Pv.05333	1.17E-47	—	—	—	—	—	Pn.12095	1.65E-47	D0vsD24
XRCC5(Ku80)	NM_021141.3	Pv.10272	1.05E-41	—	T0vsD8, T0vsD10d	—	—	—	Pn.08610	7.64E-41	—
LIG4	NM_002312.3	Pv.15195	6.24E-62	T0vsT48	T0vsD8, T0vsD10d	—	—	—	Pn.05388	1.01E-78	—

Pv11: pretreatment refers to significantly upregulated DEGs between T0 and T48; dehydration refers to significantly upregulated DEGs between T0 and D8, T0 and D10d, T48 and D8, or T48 and D10d; rehydration refers to significantly upregulated DEGs between D10d and R3, D10d and R24, or R3 and R24; genes with FDR < 0.05 were considered as DEGs. *P. vanderplanki*: dehydration refers to significantly upregulated DEGs between D0 and D24, D0 and D48, or D24 and D48; rehydration refers to significantly upregulated DEGs between D48 and R3, D48 and R24, or R3 and R24; a more than 3-fold increase in expression and an RPKM > 10 for the higher value were considered as DEGs. *P. nubifer*: dehydration refers to significantly upregulated DEGs between D0 and D24; the same criteria as for *P. vanderplanki* were used. The results for *P. vanderplanki* and *P. nubifer* is based on previous study^8^. —, no significant upregulation was detected.

### Comparative analysis among Pv11 cells, *P. vanderplanki* (a desiccation-tolerant species), *Polypedilum nubifer* (desiccation-sensitive species)

In a previous study, RNA-seq data was extracted from larvae of the desiccation-tolerant species *P. vanderplanki* during desiccation and rehydration, and from larvae of the desiccation-sensitive species *P. nubifer* during desiccation^8^. A comparative analysis of the RNA-seq data revealed upregulation of genes encoding late embryogenesis abundant proteins (LEAs), TRXs, PIMTs, haemoglobins (Hbs), aquaporins (Aqps), and various enzymes that accelerate the metabolism of trehalose in the larvae of *P. vanderplanki* compared with in the larvae of *P. nubifer*. To build on these previous results, we comparatively analysed the differential expression of these previously reported genes and those we identified in our differential gene expression analysis.

First, we analysed the differential expression of genes encoding LEAs, TRXs, PIMTs, Hbs, Aqps, and four enzymes related to the metabolism of trehalose (Table 3, Supplementary Data 4). Some genes encoding LEAs, TRXs, PIMTs, and trehalase, which is an enzyme that breaks down trehalose, were significantly upregulated in Pv11 cells. In contrast, none of the genes encoding Hbs, Aqps, or trehalose phosphate synthase (TPS) were significantly upregulated in Pv11 cells. In addition, fewer genes encoding LEAs, TRXs, and PIMTs were significantly upregulated in Pv11 cells compared with in *P. vanderplanki* larvae.

**Table 3:** Numbers of differentially expressed genes with sequence similarity to previously reported genes associated with desiccation tolerance in *P. vanderplanki*.

Type of gene	Pv11			*P. vanderplanki*		*P. nubifer*
	Pretreatment	Dehydration	Rehydration	Dehydration	Rehydration	Dehydration
LEA	3/23	5/23	0/23	20/23	0/23	0/0
TRX	3/25	7/25	1/25	20/25	0/25	0/3
PIMT	3/14	6/14	1/14	12/14	1/14	0/1
Hb	0/32	0/32	0/32	8/32	23/32	3/25
Aqp	0/5	0/5	0/5	2/5	2/5	1/5
Trehalose Synthesis	1/4(TREH)	1/4(TREH)	0/4	2/4(TPS,TREH)	0/4	0/4

LEA, late embryogenesis abundant proteins; TRX, thioredoxins; PIMT, L-isoaspartyl methyltransferase; Hb, haemoglobin; Aqp, aquaporin; Trehalose synthesis, four enzymes that accelerate trehalose synthesis. Column headings are described in Table 2. The left and right number in each cell refers to the number of DEGs and identified family genes, respectively. TPS, trehalose phosphate synthase; TREH, trehalose.

Next, we focused on the genes that are most likely to be important for desiccation tolerance–Pv.03555 (NMD), Pv.06995 (GST), Pv.11397 (UGT), Pv.04950 (carboxylesterase), Pv.08737 (ATP-dependent RNA helicase), and Pv.12163 (DEAD-box ATP-dependent RNA helicase). We found that no genes in *P. nubifer* had sequence similarity to these genes (Table 4, BLASTN E-value < 1.0E-05). With respect to the differential expression between Pv11 cells and *P. vanderplanki* larvae, Pv.03555 and Pv.04950 showed significant upregulation in *P. vanderplanki* larvae but the others showed no significant difference of expression.

**Table 4:** Differentially expressed genes with sequence similarity to genes significantly upregulated in pretreatment in Pv11 cells.

Gene Name	Pv Gene ID	Pv11			*P. vanderplanki*		*P. nubifer*
		Pretreatment	Dehydration	Rehydration	Dehydration	Rehydration	Dehydration
NMD	Pv.03555	T0vsT48	T0vsD8, T0vsD10d	—	—	D48vsR3	NA
GST	Pv.06995	T0vsT48	—	—	—	—	NA
UGT	Pv.11397	T0vsT48	—	—	—	—	NA
Carboxylesterase	Pv.04950	T0vsT48	T0vsD10d	—	—	D48vsR24, R3vsR24	NA
ATP-dependent RNA helicase	Pv.08737	T0vsT48	T0vsD10d	—	—	—	NA
DEAD box ATP-dependent RNA helicase	Pv.12163	T0vsT48	T0vsD8, T0vsD10d	—	—	—	NA

The column contents are the same as in Table 2. NA, sequence similarity not detected (BLASTN E-value < 1.0E-15); NMD, nonsense-mediated mRNA decay protein; GST, glutathione S-transferase; UGT, UDP-glucuronosyltransferase.

Our differential expression analysis revealed that HR, NER, and NHEJ are potentially important DNA repair systems for Pv11 cells during desiccation and rehydration. *P. nubifer* possessed genes with sequence similarity to those associated with HR, NER, and NHEJ; however, no genes showed significant upregulation during desiccation (Table 2, Supplementary Data 3). In contrast, genes associated with HR and NER were significantly upregulated in *P. vanderplanki* larvae. No genes associated with NHEJ were significantly upregulated in *P. vanderplanki* larvae. Thus, although DNA repair systems are likely an important part of desiccation tolerance both in Pv11 cells and *P. vanderplanki* larvae, the systems used in Pv11 cells and *P. vanderplanki* larvae are slightly different.

## Discussion

### Comparison of the desiccation tolerance system among Pv11 cells and other desiccation-tolerant species

Our comparative analysis of differential expression among Pv11 cells, *P. vanderplanki* larvae, and *P. nubifer* larvae revealed differences and similarities in gene expression between these experimental models. Among genes encoding LEAs, TRXs, PIMTs, Hbs, Aqps, and enzymes that catalyse the synthesis of trehalose, some of those encoding LEAs, TRXs, PIMTs, and trehalase, but not Hbs, Aqps, and TPS, were significantly upregulated during pretreatment and desiccation in Pv11 cells. Pv11 cells are cultured cells derived from egg masses of *P. vanderplanki*^9^. However, the embryonic cell type from which Pv11 cells were derived has not been clarified. Previous research has revealed that the fat body in *P. vanderplanki* larvae synthesizes trehalose, which protects the cells from desiccation^25,26^. Pv11 cells are unable to induce upregulation of TPS, which catalyses the reaction to synthesize trehalose. Indeed, TPS is predominantly expressed in the fat body^27^. Therefore, the properties of Pv11 cells should be different to those of fat body cells. In addition, Pv11 cells are unable to tolerate desiccation without treatment with culture medium containing 600 mM trehalose for 48 h^10^, which could be due to loss of the capacity to upregulate TPS expression. Some genes encoding LEAs, TRXs, and PIMTs were significantly upregulated in Pv11 cells; however, the number of these genes was lower than that of genes upregulated in *P. vanderplanki* larvae.

Focusing on the genes encoding LEAs, TRXs, and PIMTs that were significantly upregulated in *P. vanderplanki* larvae but not in Pv11 cells, some of the genes encoding these proteins were highly expressed in T0 (Supplementary Fig. S13). This could be because Pv11 cells are stimulated by salinity stress in normal culture with IPL-41 medium, which contains a high amount of sodium ions compared with other conventional insect media, such as MGM-433 and Grace’s medium. Previous research has shown that various anhydrobiosis-related genes in *P. vanderplanki* larvae are upregulated by sodium ion stress^9,25,28,29^. Thus, the Pv11 cells could have already been stimulated by salt stress during the initial treatment (T0) and the protein expression steadily increased up to T48. This could explain why the number of DEGs relative to T0 was lower compared with that in *P. vanderplanki* larvae.

We showed that during the rehydration process, the HR and NER DNA repair systems were likely active in Pv11 cells and *P. vanderplanki* larvae, which is consistent with previous research that has demonstrated that HR is active during rehydration in *P. vanderplanki* larvae and desiccation-tolerant tardigrade species^30,31^. In contrast, we found that the NHEJ DNA repair system could be active only in Pv11 cells. NHEJ directly religates broken double-strand DNA^32,33^. NHEJ is also known to repair DNA faster than HR^34^. Based on these previous studies and our finding that NHEJ could be only activated in Pv11 cells, we infer that Pv11 cells require a more sensitive response to DNA damage during the desiccation phase than do *P. vanderplanki* larvae.

In comparison with other desiccation-tolerant species, such as the tardigrade *Hypsibius dujardini* and dauer larvae of the nematode *C. elegans*, the number of DEGs at entry into anhydrobiosis in Pv11 cells was much lower (*H. dujardini*, 1422 DEGs^35^; *C. elegans*, 9555 DEGs^17^). Again, this could be because genes that are important for desiccation tolerance in Pv11 cells were stimulated by salinity stress in IPL-41 medium and the differential expression of these genes was hidden by the experimental conditions. Another possibility is that Pv11 cells are single cells and so more complex systems of desiccation tolerance that involve a variety of proteins, such as TPS, have been lost, and so these genes were not upregulated in Pv11 cells. However, Pv11 cells do not lose the ability to proliferate after rehydration; therefore, they could be an ideal model to investigate the simplest mechanism of desiccation tolerance.

Moreover, some desiccation tolerant species that massively accumulate trehalose as a protectant, such as *C. elegans*, *S. cerevisiae* and brine shrimp, *Artemia franciscana*, are to use LEA proteins and thioredoxins for desiccation tolerance^17,36–39^. In this study, we showed that the upregulation of genes encoding some LEA proteins and thioredoxins in Pv11 cells was caused by pretreatment and desiccation. Considering that Pv11 cells are derived from egg masses of *P. vanderplanki*, which uses trehalose as a desiccation-protectant, we inferred that LEA proteins and thioredoxins are the highly conserved components of desiccation tolerant system with trehalose synthesis in these species.

### Suggested model

Treatment with medium containing sufficient trehalose is essential for Pv11 cells to induce desiccation tolerance; Pv11 cells treated with the culture medium containing 443 mM trehalose do not survive, but with an increase to 600 mM, the survival rate increases to 16%^10^. Trehalose is a compatible solute and protects the cell membrane and intracellular biological molecules^3,4^. The detection of 384 DEGs between T0 and T48 indicates that trehalose not only directly protects biological molecules but also should regulate many genes for desiccation tolerance in Pv11 cells. The accumulation of trehalose in *P. vanderplanki* larvae starts just after the onset of desiccation and water content is steady at that time^25^. We assumed that treatment of Pv11 cells with trehalose corresponds to this time span and various genes needed to acquire desiccation tolerance are upregulated in *P. vanderplanki* larvae.

Various stress response genes (Pv.03555 and Pv.06695), genes for proteins that eliminate harmful chemicals (Pv.11397 and Pv.04950), and TRX genes (Pv.00404, Pv.06747, Pv.07504, and Pv.07510) were significantly upregulated in T48 in comparison with T0. The known biological functions of similar genes suggest that all these genes likely have a role in protecting cells from the harmful effects of desiccation.

GO analysis of DEGs between T0 and D8, T0 and D10d, T48 and D8, and T48 and D10d showed that 7 genes encoding PIMTs and 2 genes encoding HSPs were significantly upregulated in Pv11 cells in desiccation phase. These genes are key genes of desiccation tolerance in *P. vanderplanki* larvae^11,12^. Focusing on downregulated genes in desiccation, many genes encoding ribosomal proteins were significantly downregulated in D10d. This result could be because Pv11 cells repress the translation of proteins to reduce energy consumption in the desiccation phase. In the desiccation phase, we detected DEGs with GO:0005484 (SNAP receptor activity) between T0 and D10d, and DEGs with GO:0006376 (mRNA splice site selection) between T48 and D8. The relationships between genes with these GOs and desiccation tolerance has not been previously reported in a desiccation-tolerant species. Therefore, these genes could be involved in a unique system of desiccation tolerance in Pv11 cells.

In the rehydration process, Pv.07646, which has sequence similarity to the DNA repair gene Rad16, was specifically upregulated in R3. Moreover, our differential expression analysis of genes related to the HR, NER, MMR, and NHEJ DNA repair systems showed that genes associated with HR and NHEJ were significantly upregulated in the desiccation phase, and those associated with NER were upregulated in the desiccation and rehydration phases. HR and NHEJ are double-strand DNA break repair systems and NER is a single-strand DNA break repair system^23,24^. That means double strand DNA breaking repair systems, HR and NHEJ, are active in the desiccation phase and single strand DNA breaking repair system, NER, is active in the rehydration phase in Pv11 cells. These results show that the DNA repair system could be used properly in the desiccation phase and the rehydration phase in Pv11 cells.

## Methods

### CAGE RNA preparation, Illumina Hiseq-2500 sequencing, and data preprocessing

Pv11 cells were maintained in IPL-41 medium supplemented with 10% FBS (T0) and were incubated in 600 mM trehalose solution containing 10% IPL-41 medium for 48 h (T48). The cells were desiccated in a desiccator (relative humidity, <10%) for 8 h (D8) or 10 days (D10d). After D10d, the samples were rehydrated with IPL-41 containing 10% FBS for 3 h (R3) or 24 h (R24). Samples were prepared in biological triplicate. Total RNA was extracted with a RNAiso-Plus kit (TaKaRa, Shiga, Japan) and purified with a NucleoSpin RNA kit (Macherey-Nagel, Düren, Germany). RNA quality was assessed with a Bioanalyzer (Agilent Technologies, Santa Clara, CA, USA), and RNA was quantified with a Qubit 2.0 fluorometer (Thermo Fisher, Waltham, MA, USA). First-strand cDNAs were synthesized on the 5′-ends of capped RNAs attached to CAGE bar code tags. A CAGE library was prepared as described previously^40^. CAGE tags were sequenced with a HiSeq-2500 (Illumina, San Diego, CA, USA). The reads were preprocessed by removing (a) the first 3 bases from the 5′-end considering it could be capped, (b) the first 7 bases from the 3′-end if they were contiguous adenine residues because it could be a poly-A structure, and (c) reads containing more than 2 N bases because mapping such reads to the genome could result in false positives (Supplementary Table S5). The remaining reads were mapped to the genome of *P. vanderplanki* (midgeBase: http://150.26.71.110/midgebase/) using the qAlign function in the QuasR package in Bioconductor^41^. The mapped reads were counted using the qCount function in the QuasR package.

### DEG analysis

The read count data of each sample were revised to tags per million by using the iterated DEG Eliminated Strategy (iDEGES) method (TMM-(edgeR-TMM)_3_)^42^. Using the revised data, DEGs were detected by edgeR^43^ when the FDR calculated by the Benjamini-Hochberg method^44^ was less than 0.05. The differential expression of genes for each pair of samples was plotted as an M-A plot^45^. The horizontal axis of the M-A plot indicates the average expression of a gene across two groups (samples G1 and G2).

### Heat map and hierarchical clustering

The tags per million of DEGs among all samples were visualized as a heat map (CRAN: heatmap.2^46^). The similarity of the expression pattern between samples or genes was analysed by hierarchical clustering (CRAN: hclust^47^). Euclidean distance and a complete linkage method were used.

### Gene Ontology annotation and analysis by Fisher’s exact test

To assess the similarity of primary structures, BLAST searches of the non-redundant database were performed using Blast2GO^48^ with the BLASTX option and an E-value threshold of 1.0E-05. GO mapping and annotation of *P. vanderplanki* genes were performed on the basis of the BLASTX results with the thresholds of CutOff and GO level weighting set at 30 and 5, respectively (Supplementary Data 5). GO analysis was performed using Fisher’s exact test to detect enriched GO terms in the annotations^47^. FDR was calculated by the Benjamini-Hochberg method using the *p*-value from Fisher’s exact test. GOs with FDR < 0.05 were selected. GOs with similar functions were gathered by REVIGO^49^. The method of semantic similarity calculation was set up with the Rel method^50^ with the default threshold of 0.7. The selected GOs were considered enriched.

### Domain structure analysis

The similarity of protein domains of gene products registered in the Conserved Domain Database^51^ was evaluated by using a multiple sequence alignment viewer (MSAViewer: https://www.ncbi.nlm.nih.gov/projects/msaviewer/)^52^. Designations are described in Supplementary Fig. S5.

### Comparative analysis for *P. vanderplanki* and *P. nubifer*

The transcriptome data of *P. vanderplanki* larvae and *P. nubifer* larvae used in this study were based on a previous study^8^; a more than three fold increase in expression and reads per kilobase of exon per million mapped sequence reads (RPKM) greater than 10 for the higher value were considered to indicate significant upregulation.

### RT qPCR

D10d and R3 samples were prepared as in CAGE-seq RNA preparation. Cells were homogenized by ultrasound. Total RNA was extracted using a ReliaPrep RNA Tissue Miniprep System and quantified using a QuantiFluor ssDNA System (both from Promega Corporation, Madison, WI, USA). RNA was reverse transcribed into cDNA with a Transcriptor First Strand cDNA Synthesis Kit (Roche Applied Science, Mannheim, Germany) using an anchored oligo (dT) random hexamer primer (600 pmol/*μ*L) for 60 min at 60 °C and 5 min at 85 °C. cDNA was then immediately placed on ice, quantified using the QuantiFluor ssDNA System, and diluted with MilliQ water to contain 10 ng of cDNA based on the quantification result. qPCR was performed in a CFX96 Real-Time PCR Detection System (Bio-Rad, Hercules, CA, USA) for 30 s at 95 °C and 41 cycles of 5 s at 95°C and 30 s at 62 °C. Each qPCR mixture contained 10 *μ*L of SYBR Premix Ex Taq (Tli RNaseH Plus) (2×) (TaKaRa), 10 ng of cDNA, and 0.2 *μ*M each forward and reverse primers, supplemented with MilliQ water to 20 *μ*L. The primers used for Pv.07646 were 5′-TGAGACAAGACGAGCCAGATG-3′ (forward) and 5′-CCAAATGAGGAGCGGAATG-3′ (reverse). A control reaction without the template was also performed. Fold change of gene expression in R3 relative to D10d was calculated as ${2}^{{C}_{t}(D10d)-{C}_{t}(R\mathrm{3)}}$, where *C*_*t*_ is the threshold cycle. The significant change between D10d and R3 was tested by Welch’s t-test, and *p*-value < 0.05 was considered significant.

## Electronic supplementary material

Supplementary Figure and Table

Supplementary Data 1

Supplementary Data 2

Supplementary Data 3

Supplementary Data 4

Supplementary Data 5

## Data Availability

CAGE-seq data of the mRNA reported are available in the DNA Data Bank of Japan Sequenced Read Archive under the accession number DRA007433. All other data generated or analysed during this study are included in this published article and its Supplementary Information files.
