# Supplementary material for: Transcriptome analysis of the anhydrobiotic cell line Pv11 infers the mechanism of desiccation tolerance and recovery
Source: Sci Rep. 2018 Dec 18;8:17941. doi: 10.1038/s41598-018-36124-6 (PMC6298976; doi:10.1038/s41598-018-36124-6)
Supplement: Supplementary file 1 — Supplementary Figure and Table [file 41598_2018_36124_MOESM1_ESM.pdf]

Transcriptome analysis of the anhydrobiotic cell line Pv11 infers the mechanism of desiccation tolerance and recovery

Takahiro G Yamada[1], Yoshitaka Suetsugu[2,†], Ruslan Deviatiiarov[3], Oleg Gusev[3,4], Richard Cornette[2], Alexander Nesmelov[3], Noriko Hiroi[5], Takahiro Kikawada[2,6,*], and Akira Funahashi[1,*]

[1]Department of Biosciences and Informatics, Keio University, Yokohama, Kanagawa, 223-8522, Japan
[2]Institute of Agrobiological Sciences, National Agriculture and Food Research Organization (NARO), Tsukuba, Ibaragi, 305-8634, Japan
[3]Kazan Federal University, Kazan, Tatarstan, 420008, Russia
[4]RIKEN, Yokohama, Kanagawa, 230-0045, Japan
[5]Faculty of Pharmaceutical Science, Sanyo-Onoda City University, Sanyo-Onoda, Yamaguchi, 756-0884, Japan
[6]Graduate School of Frontier Sciences, The University of Tokyo, Kashiwa, Chiba, 277-8562, Japan
[*]kikawada@affrc.go.jp and funa@bio.keio.ac.jp
[†]Deceased

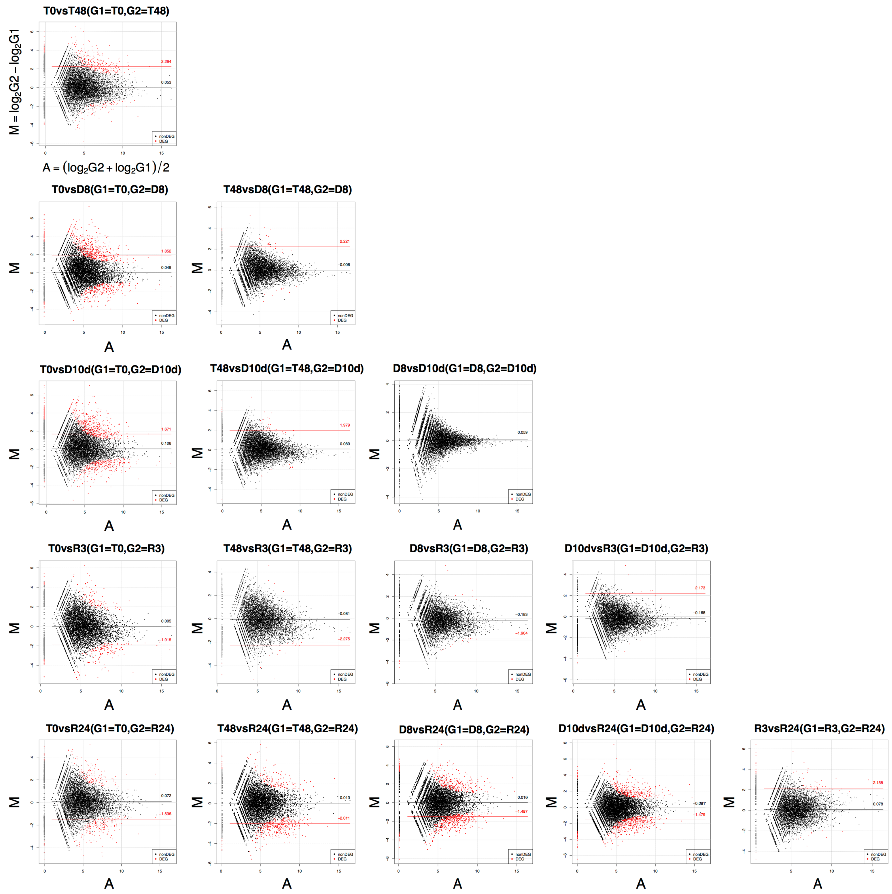

Supplementary Figure S1. M-A plots of differentially expressed genes (DEGs) for each pair of samples. The M value indicates the log ratio (sample group 2 (G2) relative to sample group 1 (G1), $log_2G2 - log_2G1$). The A value indicates the average expression of a gene in samples G1 and G2 ($(log_2G2 + log_2G1)/2$). The median M values are indicated by black lines for non-DEGs and red lines for DEGs.

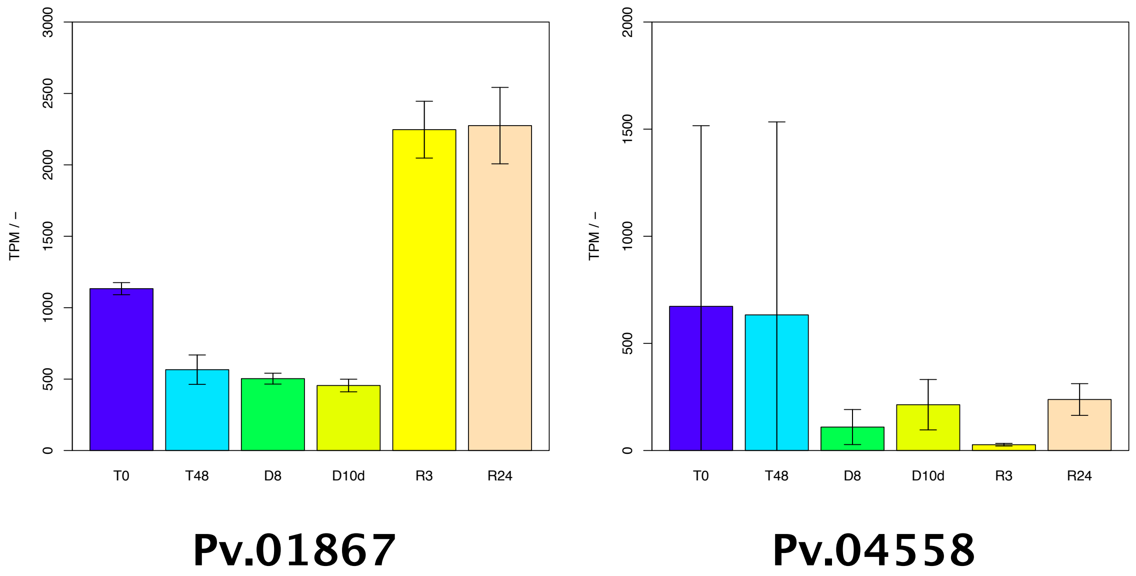

Supplementary Figure S2. Expression of Pv.01867 and Pv.04558. Horizontal axis shows sample names. TPM, tags per million. T0, control Pv11 cells. T48, trehalose treatment for 48 h. D8, desiccation for 8 h. D10d, desiccation for 10 days. R3, rehydration of D10d cells for 3 h. R24, rehydration of D10d cells for 24 h. Data are mean ± SD, n=3.

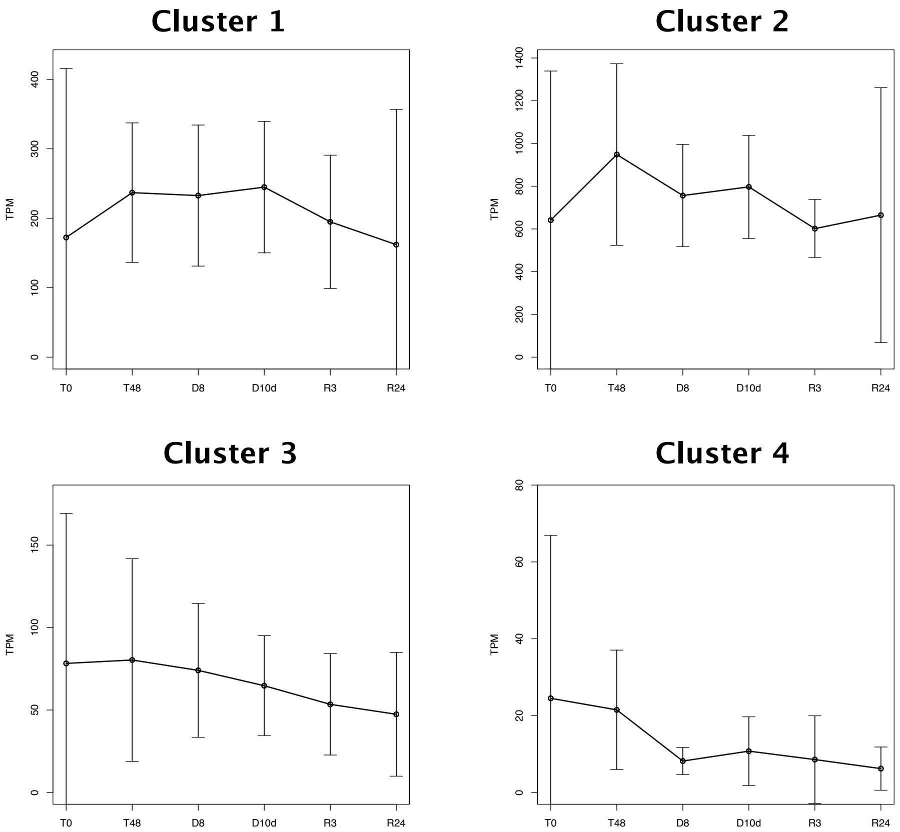

Supplementary Figure S3. Time course of the expression of DEGs annotated by GO:0008152 (metabolic process) (mean $\pm$ SD).

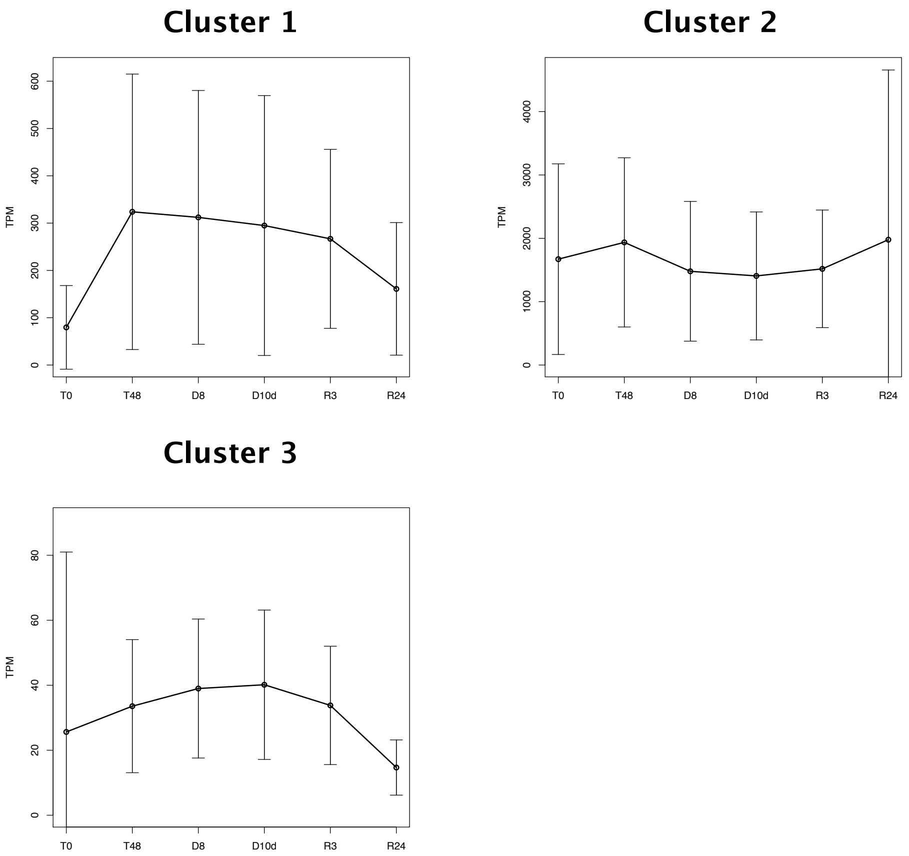

Supplementary Figure S4. Time course of the expression of DEGs annotated by GO:0055114 (oxidation-reduction process) (mean ± SD).

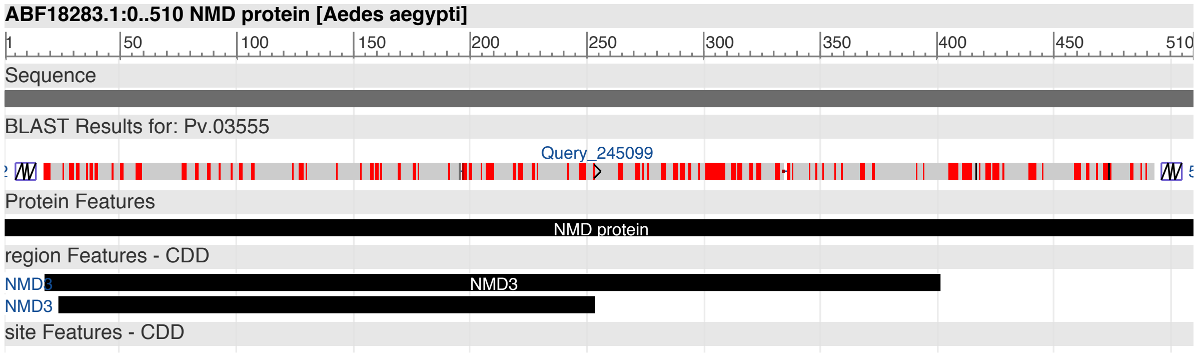

Supplementary Figure S5. Alignment of Pv.03555 with nonsense-mediated mRNA decay (NMD) protein. The visualization was created by using MSAViewer (https://www.ncbi.nlm.nih.gov/projects/msaviewer/). Sequence: sequence of the subject gene; Blast Results: alignment results between the *P. vanderplanki* gene and the subject gene by BLASTX; Protein Features, specific regions of the proteins; region Features, specific regions of the subject gene; site Features, specific sites of the subject gene. Under BLAST Results, gaps are represented as thick, dark-red, horizontal lines; insertions as black vertical bars and black arrowheads; and aligned regions as grey bars (match regions) or red bars (mismatch regions). The regions and sites of the subject gene that overlapped with those of the aligned *P. vanderplanki* gene were considered present.

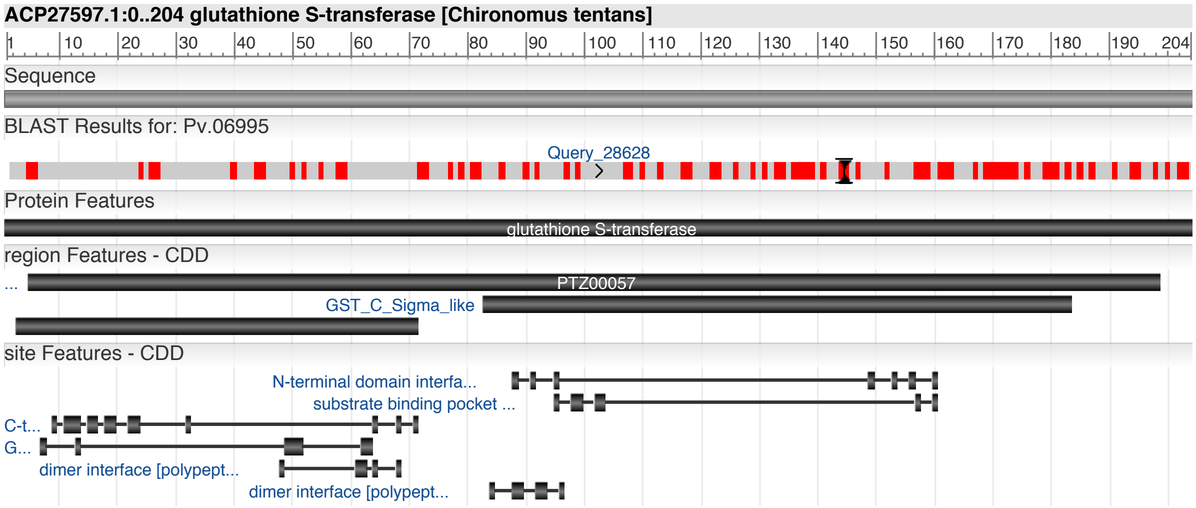

Supplementary Figure S6. Alignment of Pv.06995 with glutathione S-transferase.

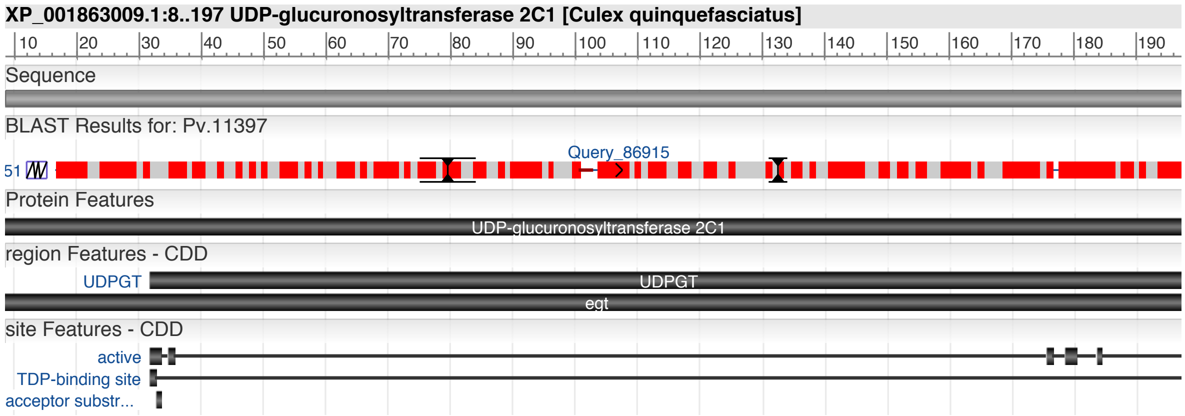

Supplementary Figure S7. Alignment of Pv.11397 with UDP-glucuronosyltransferase 2C1.

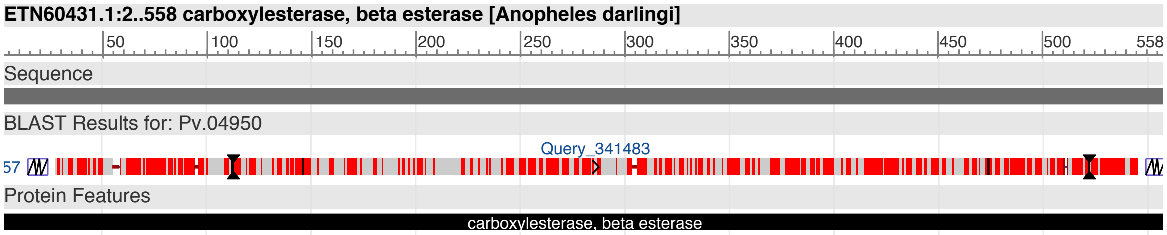

Supplementary Figure S8. Alignment of Pv.04950 with carboxylesterase, beta esterase.

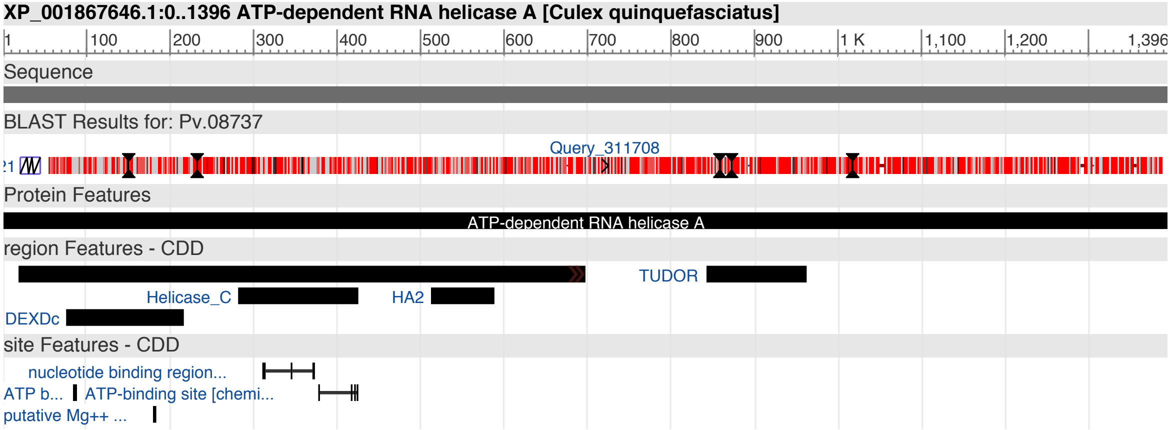

Supplementary Figure S9. Alignment of Pv.08737 with ATP-dependent RNA helicase A.

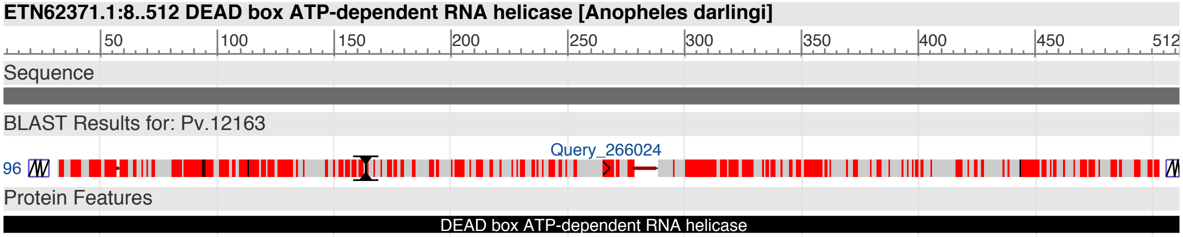

Supplementary Figure S10. Alignment of Pv.12163 with DEAD-box ATP-dependent RNA helicase.

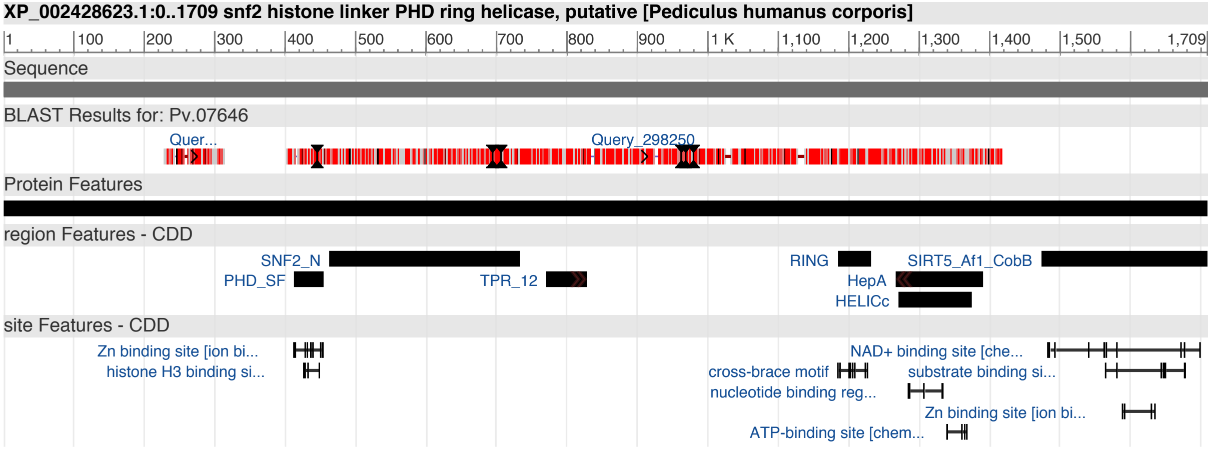

Supplementary Figure S11. Alignment of Pv.07646 with SNF2 histone linker PHD-ring helicase, annotated as putatively involved in desuccinylation.

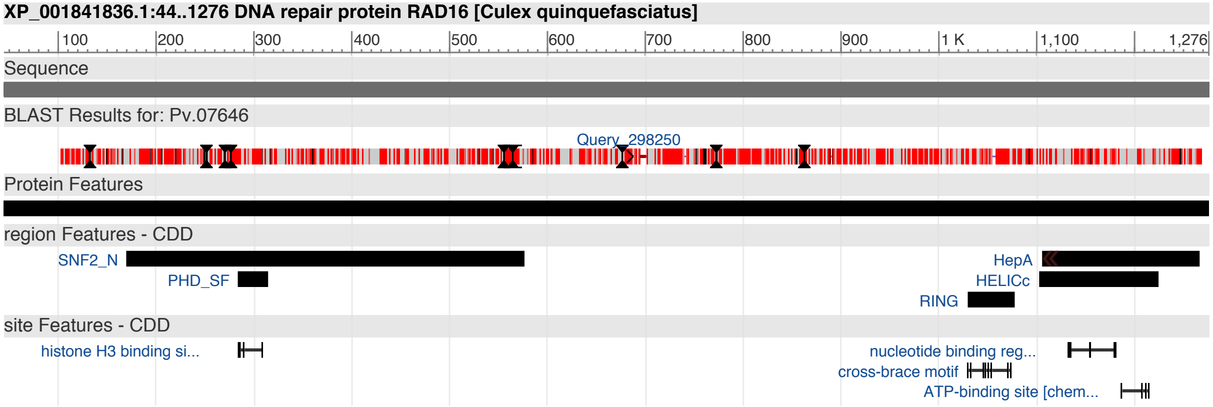

Supplementary Figure S12. Alignment Pv.07646 with the DNA-repair protein RAD16.

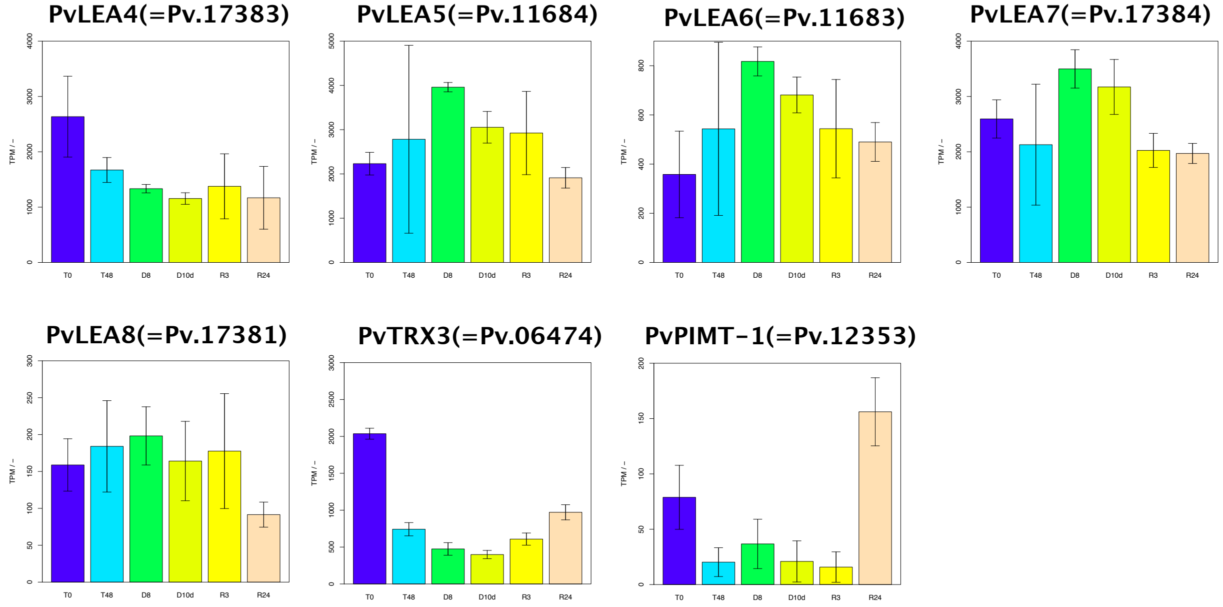

Supplementary Figure S13. Time course expression of the genes encoding late embryogenesis abundant proteins (LEA), thioredoxin (TRX), and protein L-isoaspartyl methyltransferase (PIMT) genes.

Supplementary Table S1. DEGs between D10d and R3.

	PvGene ID	Gene Identifier	BLAST first hit gene	E.value
Upregulated in R3	Pv.04891	194753680	GF12734	1.71E-9
	Pv.07646	170027903	DNA repair protein RAD16	0.0
	Pv.01911	765338102	AAEL005023-PA	1.17E-56
	Pv.00327	170050025	conserved hypothetical protein	2.20E-61
	Pv.08602	668455465	AGAP001139-PA-like protein	3.67E-102
	Pv.01867	157136676	AAEL013609-PA, partial	0.0
	Pv.03099		No Hits	
Downregulated in R3	Pv.04558	170036404	conserved hypothetical protein	2.62E-107
	Pv.08152		No Hits	
	Pv.11244	157128927	AAEL011272-PA, partial	2.15E-126
	Pv.13360	170052229	FAM96B	7.93E-77
	Pv.05172	157104834	AAEL004151-PB	4.96E-25
	Pv.13909	751792306	PREDICTED: Y+L amino acid transporter 2	0.0
	Pv.10501	170050626	cyclin b	1.49E-109

Supplementary Table S2. Annotated gene ontology to the DEGs identified between D10d and R3.

Pv Gene ID	Annotated GO	GO Description
Pv.04891	GO:0016301	kinase activity
	GO:0016310	phosphorylation
Pv.00327	GO:0000166	nucleotide binding
	GO:0003676	nucleic acid binding
	GO:0016779	nucleotidyltransferase activity
	GO:0046872	metal ion binding
	GO:0008152	metabolic process
Pv.08602	GO:0005634	nucleus
	GO:0005762	mitochondrial large ribosomal subunit
	GO:0003676	nucleic acid binding
	GO:0003735	structural constituent of ribosome
	GO:0008270	zinc ion binding
	GO:0006412	translation
Pv.07646	GO:0000786	nucleosome
	GO:0005634	nucleus
	GO:0003677	DNA binding
	GO:0004386	helicase activity
	GO:0004842	ubiquitin-protein transferase activity
	GO:0005524	ATP binding
	GO:0008270	zinc ion binding
	GO:0016874	ligase activity
	GO:0036054	protein-malonyllysine demalonylase activity
	GO:0036055	protein-succinyllysine desuccinylase activity
	GO:0070403	NAD+ binding
	GO:0000209	protein polyubiquitination
	GO:0006334	nucleosome assembly
	GO:0022008	neurogenesis
	GO:0036047	peptidyl-lysine demalonylation
	GO:0036049	peptidyl-lysine desuccinylation
Pv.01911	GO:0005794	Golgi apparatus
	GO:0016021	integral component of membrane
	GO:0003676	nucleic acid binding
	GO:0008270	zinc ion binding
	GO:0019706	protein-cysteine S-palmitoyltransferase activity
	GO:0006888	ER to Golgi vesicle-mediated transport
	GO:0018345	protein palmitoylation
Pv.04558	GO:0005826	actomyosin contractile ring
	GO:0016324	apical plasma membrane
	GO:0016328	lateral plasma membrane
	GO:0030478	actin cap
	GO:0045178	basal part of cell
	GO:0045179	apical cortex
	GO:0005089	Rho guanyl-nucleotide exchange factor activity
	GO:0019992	diacylglycerol binding
	GO:0046872	metal ion binding
	GO:0007015	actin filament organization
	GO:0007277	pole cell development
	GO:0007349	cellularization
	GO:0007370	ventral furrow formation
	GO:0007374	posterior midgut invagination
	GO:0007375	anterior midgut invagination
	GO:0007377	germ-band extension
	GO:0016476	regulation of embryonic cell shape
	GO:0030589	pseudocleavage involved in syncytial blastoderm formation
	GO:0031532	actin cytoskeleton reorganization
	GO:0035025	positive regulation of Rho protein signal transduction
	GO:0035277	spiracle morphogenesis, open tracheal system
	GO:0035556	intracellular signal transduction
	GO:0038032	termination of G-protein coupled receptor signaling pathway
	GO:0043547	positive regulation of GTPase activity
	GO:0050770	regulation of axonogenesis
	GO:0070252	actin-mediated cell contraction
	GO:0090254	cell elongation involved in imaginal disc-derived wing morphogenesis
Pv.01867	GO:0005886	plasma membrane
	GO:0012505	endomembrane system
	GO:0015279	store-operated calcium channel activity
	GO:0048763	calcium-induced calcium release activity
	GO:0002115	store-operated calcium entry
	GO:0007155	cell adhesion
	GO:0007476	imaginal disc-derived wing morphogenesis
	GO:0022416	chaeta development
	GO:0032237	activation of store-operated calcium channel activity
	GO:0051533	positive regulation of NFAT protein import into nucleus
Pv.11244	GO:0017146	NMDA selective glutamate receptor complex
	GO:0004972	NMDA glutamate receptor activity
Pv.13360	GO:0005737	cytoplasm
	GO:0007059	chromosome segregation
	GO:0016226	iron-sulfur cluster assembly
Pv.05172	GO:0022625	cytosolic large ribosomal subunit
	GO:0003682	chromatin binding
	GO:0003735	structural constituent of ribosome
	GO:0006412	translation
Pv.13909	GO:0016021	integral component of membrane
	GO:0015171	amino acid transmembrane transporter activity
	GO:0003333	amino acid transmembrane transport
	GO:0060356	leucine import
Pv.10501	GO:0000775	chromosome, centromeric region
	GO:0019908	nuclear cyclin-dependent protein kinase holoenzyme complex
	GO:0045495	pole plasm
	GO:0051233	spindle midzone
	GO:0072686	mitotic spindle
	GO:0016538	cyclin-dependent protein serine/threonine kinase regulator activity
	GO:0000086	G2/M transition of mitotic cell cycle
	GO:0000281	mitotic cytokinesis
	GO:0000920	cell separation after cytokinesis
	GO:0001700	embryonic development via the syncytial blastoderm
	GO:0006974	cellular response to DNA damage stimulus
	GO:0007079	mitotic chromosome movement towards spindle pole
	GO:0008608	attachment of spindle microtubules to kinetochore
	GO:0035186	syncytial blastoderm mitotic cell cycle
	GO:0035561	regulation of chromatin binding

Supplementary Table S3. Differentially expressed genes between T0 and T48 with GO:0008152 (metabolic process). PREDICTED, initial RefSeq record status meaning the annotation is only predicted by automated BLAST analysis and has not been validated experimentally.

Cluster Num.	Pv Gene ID	Gene Identifier	BLAST First Hit Gene	E-value
Cluster 1	Pv.00012	755889065	PREDICTED: uncharacterized protein LOC101899881 isoform X2	3.5155E-21
	Pv.00503	170031381	conserved hypothetical protein	0.0
	Pv.01170	195109112	GI23236	0.0
	Pv.01614	557775558	PREDICTED: tubulin beta-1 chain	0.0
	Pv.01749	158296880	AGAP008252-PA	0.0
	Pv.01933	755849759	PREDICTED: GTP-binding protein 1-like	0.0
	Pv.03555	94468868	NMD protein	0.0
	Pv.05750	347969792	AGAP003372-PB	8.73E-102
	Pv.06865	157120582	AAEL009068-PA	0.0
	Pv.07614	568250312	transport and golgi family organization	2.44E-75
	Pv.07843	668455678	hypothetical protein ZHAS_00011763	4.90E-162
	Pv.08737	170064729	ATP-dependent RNA helicase A	0.0
	Pv.11162	668458368	AGAP004610-PA-like protein	0.0
	Pv.12163	568253176	DEAD box ATP-dependent RNA helicase	3.32E-146
	Pv.13802	170049985	conserved hypothetical protein	2.60E-86
	Pv.15605	157107882	AAEL014906-PA, partial	2.81E-76
	Pv.17087	817062658	PREDICTED: E3 ubiquitin-protein ligase rnf146-like	1.19E-32
Cluster 2	Pv.02231	751789335	PREDICTED: 5-oxoprolinase	0.0
	Pv.06995	227343489	glutathione S-transferase	5.64E-87
	Pv.10576	347969074	AGAP003018-PA	0.0
	Pv.12167	157110933	AAEL000854-PA	9.32E-141
Cluster 3	Pv.02391	749772182	PREDICTED: abhydrolase domain-containing protein 4 isoform X1	9.38E-112
	Pv.06862	568250143	Elongator complex protein 3	0.0
	Pv.08047	332373216	unknown	8.53E-36
	Pv.11397	170054183	UDP-glucuronosyltransferase 2C1	8.03E-30
	Pv.12035	157117142	AAEL007889-PA	0.0
	Pv.13081	157136208	AAEL003433-PA	0.0
	Pv.13347	557763449	PREDICTED: E3 ubiquitin-protein ligase Topors isoform X2	3.02E-56
	Pv.16072	668446285	AGAP008326-PA-like protein	1.05E-145
Cluster 3	Pv.04950	568250792	carboxylesterase, beta esterase	1.67E-138
	Pv.06833	589278470	hypothetical protein TREMEDRAFT_39272	7.49E-26
	Pv.13234	668450732	hypothetical protein ZHAS_00007358	1.72E-28

Supplementary Table S4. Differentially expressed genes between T0 and T48 with GO:0055114 (oxidation-reduction process).

Cluster Num.	Pv Gene ID	Gene Identifier	BLAST First Hit Gene	E-value
Cluster 1	Pv.00927	646723765	hypothetical protein L798_06909	2.76E-50
	Pv.02233	157115285	AAEL001183-PA	4.90E-121
	Pv.02556	568253142	augmenter of liver regeneration	1.03E-70
	Pv.03565	170049364	cytochrome P450	2.25E-162
	Pv.03626	157123150	AAEL009396-PA	6.56E-86
	Pv.03974	157124708	AAEL001860-PA	1.28E-16
	Pv.05128	568248619	hypothetical protein AND_009968	4.30E-154
	Pv.05617	262410959	ferritin heavy chain-like protein	6.71E-100
	Pv.05897	157112413	AAEL006129-PA	9.68E-144
	Pv.08902	304367635	catalase	0.0
	Pv.10292	751781624	PREDICTED: dehydrogenase/reductase SDR family member 11-like	1.70E-66
	Pv.12828	157132840	AAEL002901-PA	3.11E-87
	Pv.12949	766940976	PREDICTED: superoxide dismutase	5.00E-64
	Pv.15455	157132518	AAEL012457-PA	1.20E-142
Cluster 2	Pv.05587	584594454	cytochrome P450 6FV2	0.0
	Pv.05872	157111212	AAEL005790-PA	0.0
	Pv.06474	528744594	thioredoxin	5.87E-91
	Pv.07012	157137514	AAEL003645-PA	1.56E-101
Cluster 3	Pv.00404	528744570	thioredoxin	1.20E-90
	Pv.01417	584594454	cytochrome P450 6FV2	0.0
	Pv.01758	157130887	AAEL002658-PB	1.04E-92
	Pv.04458	347967826	AGAP002418-PA	5.58E-129
	Pv.07504	528744568	thioredoxin	7.07E-77
	Pv.07510	528744554	thioredoxin, partial	3.38E-95
	Pv.08589	157135753	AAEL013389-PA	0.0
	Pv.10571	157118100	AAEL008160-PA	0.0

Supplementary Table S5. Preprocessing and mapping. Samples were prepared in biological triplicate. totalSequences, number of sequenced reads; tooShort, number of reads shorter than 14 base pairs; tooManyN, number of reads containing more than 2 N bases; totalPassed, tooShort and tooManyN subtracted from totalSequences; mapped, number of reads mapped to the *P. vanderplanki* genome; unmapped, number of reads not mapped to the same genome; MappingRate, proportion of mapped reads among all passed reads.

	Preprocess Result				Mapping Result		
	totalSequences	tooShort	tooManyN	totalPassed	mapped	unmapped	MappingRate
T0-Sample1	9329152	1089685	33	8239434	1682490	6556944	20.4
T0-Sample2	8618767	1642930	23	6975814	2463740	4512074	35.3
T0-Sample3	5486398	1002516	12	4483870	1517136	2966734	33.8
T48-Sample1	8249645	907419	27	7342199	1282918	6059281	17.5
T48-Sample2	4527055	923602	9	3603444	1340121	2263323	37.2
T48-Sample3	5862177	1172255	20	4689902	1747773	2942129	37.3
D8-Sample1	7162127	867901	23	6294203	1263235	5030968	20.1
D8-Sample2	5497776	914899	15	4582862	1384844	3198018	30.2
D8-Sample3	4710977	789877	19	3921081	1214370	2706711	31.0
D10d-Sample1	9786341	972550	49	8813742	1410369	7403373	16.0
D10d-Sample2	5046272	887863	13	4158396	1358339	2800057	32.7
D10d-Sample3	6176969	1146552	21	5030396	1716458	3313938	34.1
R3-Sample1	2269038	431391	3	1837644	687580	1150064	37.4
R3-Sample2	1966504	340563	6	1625935	533487	1092448	32.8
R3-Sample3	4409262	614660	6	3794596	1188580	2606016	31.3
R24-Sample1	3580658	819575	8	2761075	1228917	1532158	44.5
R24-Sample2	5913507	1330397	16	4583094	2078353	2504741	45.3
R24-Sample3	5071881	1122334	14	3949533	1752922	2196611	44.4
